# Characterization and cytotoxic effect of aqua-(2,2′,2′′-nitrilotriacetato)-oxo-vanadium salts on human osteosarcoma cells

**DOI:** 10.1007/s10534-017-0001-6

**Published:** 2017-02-15

**Authors:** Aleksandra Tesmar, Dariusz Wyrzykowski, Rafał Kruszyński, Karolina Niska, Iwona Inkielewicz-Stępniak, Joanna Drzeżdżon, Dagmara Jacewicz, Lech Chmurzyński

**Affiliations:** 1 Faculty of Chemistry, University of Gdańsk, Wita Stwosza 63, 80-308 Gdańsk, Poland; 2 Institute of General and Ecological Chemistry, Technical University of Łódź, Żwirki 36, 90-924 Łódź, Poland; 3 Department of Medical Chemistry, Medical University of Gdańsk, Dębinki 1, 80-211 Gdańsk, Poland

**Keywords:** Vanadium, Osteosarcoma cells, Antitumor activity, Crystal structure, Potentiometric titration

## Abstract

The use of protonated N-heterocyclic compound, i.e. 2,2′-bipyridinium cation, [bpyH^+^], enabled to obtain the new nitrilotriacetate oxidovanadium(IV) salt of the stoichiometry [bpyH][VO(nta)(H_2_O)]H_2_O. The X-ray measurements have revealed that the compound comprises the discrete mononuclear [VO(nta)(H_2_O)]^−^ coordination ion that can be rarely found among other known compounds containing nitrilotriacetate oxidovanadium(IV) moieties. The antitumor activity of [bpyH][VO(nta)(H_2_O)]H_2_O and its phenanthroline analogue, [phenH][VO(nta)(H_2_O)](H_2_O)_0.5_, towards human osteosarcoma cell lines (MG-63 and HOS) has been assessed (the LDH and BrdU tests) and referred to *cis*-Pt(NH_3_)_2_Cl_2_ (used as a positive control). The compounds exert a stronger cytotoxic effect on MG-63 and HOS cells than in untransformed human osteoblast cell line. Thus, the [VO(nta)(H_2_O)]^−^ containing coordination compounds can be considered as possible antitumor agents in the osteosarcoma model of bone-related cells in culture.

**Electronic supplementary material:**

The online version of this article (doi:10.1007/s10534-017-0001-6) contains supplementary material, which is available to authorized users.

## Introduction

**Fig. 1:**
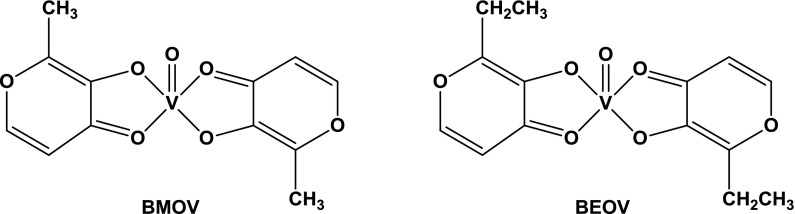
Schematic molecular structures of VO(maltolato)_2_ (BMOV) and VO(Etmaltolato)_2_ (BEOV)

Despite numerous attempts to define the role of vanadium in biological processes its impact on the functioning of higher organisms remains to be elucidated. During the last 10–15 years, progress in the chemistry of vanadium, namely in the search of its therapeutic applications has been exponential and several reviews have been published (Rehder 2013; Willsky et al. 2011; Pessoa and Tomaz 2010; Jakusch et al. 2011; Gambino 2011; Pessoa et al. 2015a, b; Kioseoglou et al. 2015; Leon et al. 2016a, b; Rehder 2017). In particular, much attention has been paid on insulin-mimetic (-enhancing) properties (Srivastava and Mehdi 2005; Marzban and McNeill 2003; Thompson et al. 2009). Among the compound tested as small molecule insulin-mimetics, or insulin-enhancers, VO(maltolato)_2_ (BMOV) (McNeill et al. 1992; Levina and Lay 2011) and VO(Etmaltolato)_2_ (BEOV) (Thompson et al. 2009) have been extensively studied (Fig. 1). BMOV and BEOV may be taken orally and both lower plasma glucose levels in streptozotocin-induced (STZ) diabetic rats (Thompson and Orvig 2006), BEOV having completed Phase I and IIa of clinical trials.

**Fig. 2:**
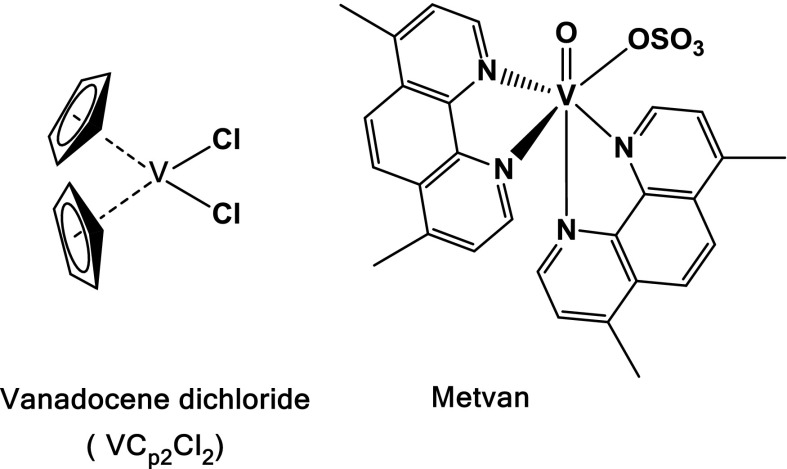
Anti-tumor vanadium coordination compounds

In recent years the anticancer properties of vanadium(IV) compounds have been noticed (Kioseoglou et al. 2015). The bis(cyclopentadienyl) dichloro-V(IV), vanadocene dichloride, [VCp_2_Cl_2_], vanadocene’s simplest derivative, as well as Metvan, V^4+^-derivative, were found to be promising anticancer drug agents (Fig. 2).

The vanadocene(IV) compound, [VCp_2_Cl_2_], was extensively studied in preclinical testing against both animal and human cancer cell lines, exhibiting a high in vitro activity (Havelek et al. 2012; Vinklarek et al. 2004; Gleeson et al. 2009; Palackova et al. 2007). Metvan induces apoptosis in different tumoral cell lines of human origin such as leukemia cells, breast cancer, ovarian, prostate and testicular cancer patients (Evangelou 2002; D’Cruz and Uckun 2002; Dong et al. 2000). The broad spectrum of anticancer activity of Metvan together with favorable pharmacodynamic features and a lack of toxicity emphasizes that this V^4+^-compound has a potential to be the first vanadium coordination compound as an alternative to the platinum-based chemotherapy (D’Cruz and Uckun 2002).

**Fig. 3:**
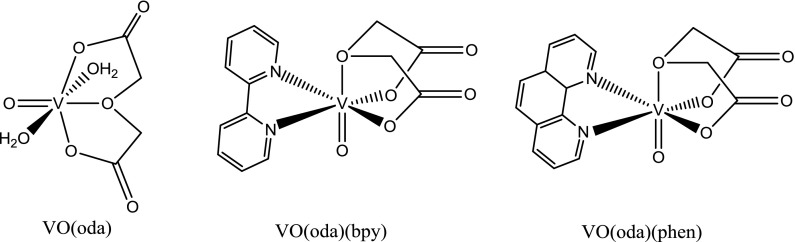
Structural formulae of VO-oda coordination compounds

Another interesting group of vanadium compounds are complexes of oxidovanadium(IV) with ligands that hold multiple donor atoms able to coordinate with metal centers. Binary and ternary oxodiacetate (oda) coordination compounds of VO^2+^, VO(oda), VO(oda)(bpy) and VO(oda)(phen), display important effects in bone related cells in culture (Fig. 3) (Rivadeneira et al. 2010).

All these compounds were tested on two osteoblast-like cell lines in culture (MC3T3E1 derived from mouse calvaria and UMR106 derived from rat osteosarcoma cells). VO(oda) caused an inhibition of a cellular proliferation in both cell lines, but the cytotoxicity was stronger in the normal (MC3T3E1) than in the tumoral (UMR106) osteoblasts. VO(oda)(phen) in the osteoblastic model caused the inhibition of the cellular proliferation in both cell lines (MC3T3E1 and UMR106), but the cytotoxicity was stronger in the normal than in the tumoral osteoblasts (León et al. 2012b). On the contrary, VO(oda)(bpy) was statistically stronger in the tumoral cells (León et al. 2012b). A nuclease activity of the three compounds (Fig. 3) revealed that the DNA cleavages caused by VO(oda)(bpy) and VO(oda) were similar, while VO(oda)(phen) showed a stronger effect. VO(oda)(phen) presented the most potent antitumor action in human osteosarcoma cells followed by VO(oda)(bpy) and then by VO(oda) according to the number of intercalating heterocyclic moieties (Yodoshi et al. 2007).

The subject of our continuous interest are polycarboxylate vanadium coordination compounds since it has been found that they are able to scavenge superoxide free radicals (O_2_^·−^) as well as protect the HT22 hippocampal neuronal cell line against an oxidative damage (Tesmar et al. 2015; Wyrzykowski et al. 2013, 2015a, b). The participation of the oxidovanadium(IV) compounds in leveling of reactive oxygen and nitrogen species (RONS) suggests that vanadium compounds can be beneficial in the treatment of several diseases and malfunctions related to RONS imbalances (Pessoa et al. 2015b). However, the main concern as regards the application of vanadium compounds as drugs is to minimalize their adverse side effects (Shukla et al. 2006). It is the crucial issue for the future use of vanadium-based drugs in medicine. For these reasons the studies on structure, physicochemical and biological properties of the vanadium compounds with a potential pharmacological ability are the subject of interest to many research groups.

Strong chelating ligands are very important in aqueous systems since they are models for trapping, transport and storage of different metallic species in living organisms (Harding et al. 1993). For this reason we have used nitrilotriacetate ions (nta) as they are known to form fairly stable complexes with oxidovanadium(IV) ions (Felcman and Fraústo da Silva 1983). In this paper, the crystal structure and physicochemical properties of the new VO^2+^-compound, namely 2,2′-bipyridinium aqua-(2,2′,2′′-nitrilotriacetato)-oxo-vanadium monohydrate, [bpyH][VO(nta)(H_2_O)]·H_2_O, is presented. Additionally, anti-proliferative and cytotoxic effects of [bpyH][VO(nta)(H_2_O)]H_2_O and its phenanthroline analogue, [phenH][VO(nta)(H_2_O)](H_2_O)_0.5_ on human osteosarcoma cell lines (MG-63 and HOS) and untransformed human osteoblast cell line (hFOB 1.19) have been assessed and compared with the properties found for cisplatin.

## Materials and methods

The reagents (Sigma-Aldrich) used for the chemical studies were of analytical grade and were used without further purification. They were as follows: VO(acac)_2_ (≥98%), nitrilotriacetic acid (H_3_nta) (≥99%), 2,2′-bipyridyl (bpy, ≥98%), NBT (nitro blue tetrazolium, 98% purity), KO_2_ (96% purity) and 18-Crown-6 (99% purity), ascorbic acid (≥99%), ABTS [2,2′-Azino-bis(3-ethylbenzothiazoline-6-sulfonic acid) diammonium salt, ≥ 98% (HPLC)] and Trolox (6-Hydroxy-2,5,7,8-tetramethylchromane-2-carboxylic acid, 98%).

### Synthesis of [bpyH][VO(nta)(H_2_O)]H_2_O

The synthesis was carried out by a method similar to that previously used for the preparation of the phenanthrolinium salt (Tesmar et al. 2015). Thus, the mixture of VO(acac)_2_ (2.65 g) and H_3_nta (1.91 g) in water (40 mL) was refluxed for ca. 0.5 h. The hot solution was filtered and cooled. To this solution, the methanolic solution of 2,2′-bipyridyl (1.56 g) was added. Then, the mixture was concentrated (in order to eliminate Hacac by an evaporation) and left for a crystallization at the room temperature. After 14 days a blue precipitate of the compound fell out. The recrystallization from hot water gave blue crystals after 7 days. The crystals of [bpyH][VO(nta)(H_2_O)]H_2_O were air-dried at the room temperature. The composition of the compound studied was established on the basis of the elemental analysis of carbon, hydrogen and nitrogen (Vario EL analyzer Cube CHNS). Anal. Calcd for [bpyH][VO(nta)(H_2_O)]H_2_O: C, 42.9%, H, 4.3%, N, 9.4%, Found: C, 42.7%, H, 4.3%, N, 9.3%. Aqueous solutions of the investigated compounds have shown a high stability, e.g. being resistant to the oxidation in air, i.e. remain unaltered (UV–Vis control) for at least 3 days.

### X-ray measurements

The blue hexagonal prism crystal of [bpyH][VO(nta)(H_2_O)]H_2_O was sealed in a glass capillary filled with helium and next it was mounted on the Bruker APEXII automatic diffractometer equipped with the CCD detector, and used for a data collection. X-ray intensity data were collected with the graphite monochromated Cu*K*_α_ (λ = 1.54178 Å) radiation at temperature 100.0(1) K, with the *ω* scan mode. The 27 s exposure time was used and reflections inside the Ewald sphere were collected up to *θ* = 72.4°. The unit cell parameters were determined from 124 strongest reflections. Details concerning the crystal data and refinement are given in Table 1. Examination of reflections on two reference frames monitored after each 20 frames measured showed no loss of the intensity during measurements. During the data reduction the Lorentz, polarization and empirical absorption (Sheldrick 2003) corrections were applied. The structure was solved by the dual-space algorithm implemented in the XT software (Sheldrick 2015a). All the non-hydrogen atoms were refined anisotropically using the full-matrix, least-squares technique on F^2^. All the hydrogen atoms were found from the difference Fourier synthesis after four cycles of an anisotropic refinement, and refined as “riding” on the adjacent atom with a geometric idealisation after each cycle of refinement and individual isotropic displacement factors equal 1.2 times the value of equivalent displacement factor of the parent methyl carbon atoms, and 1.5 times of parent oxygen or nitrogen atoms. The XL software (Sheldrick 2015b) was used for the refinement of the structure model. Atomic scattering factors were those incorporated in the computer programs. Tables of crystal data and structure refinement, anisotropic displacement coefficients, atomic coordinates and equivalent isotropic displacement parameters for non-hydrogen atoms, H-atom coordinates and isotropic displacement parameters, bond lengths and interbond angles have been deposited with the Cambridge Crystallographic Data Centre under No. CCDC1483068.

**Table 1:** Crystal and structure refinement data of [bpyH][VO(nta)(H_2_O)]H_2_O

Compound	[bpyH][VO(nta)(H_2_O)]H_2_O
Empirical formula	C_16_H_19_N_3_O_9_V
Formula weight	448.28
Crystal system, space group	Monoclinic, *P*2_1_/*n* (No.14)
Unit cell dimensions (Å, °)	*a* = 7.3532(6)
	*b* = 9.6573(13)
	*c* = 25.403(3)
	*β* = 90.678(9)
Volume (Å^3^)	1803.8(4)
Z, Calculated density (Mg/m^3^)	4, 1.651
*F*(000)	924
Crystal size (mm)	0.120, 0.116, 0.109
*θ* range for data collection (°)	3.480 to 72.401
Index ranges	−7 ≤ *h *≤ 9, −11 ≤ *k *≤ 11, −31 ≤ *1 *≤ 31
Reflections collected/unique	19203/3547 (R_*(int)*_ = 0.0241)
Completeness (%)	99.9 (to *θ* = 67°)
Data/restraints/parameters	3547/0/263
Goodness-of-fit on *F* ^2^	1.064
Final *R* indices [*I* > 2σ(*I*)]	*R*1 = 0.0255, *wR* ^2^ = 0.0674
R indices (all data)	*R*1 = 0.0255, *wR* ^2^ = 0.0674
Largest diff. peak and hole (e·Å^−3^)	0.394, −0.419

### IR spectra

The IR spectra were recorded on the BRUKER IFS 66 spectrophotometer in a KBr pellet over the 4400–650 cm^−1^ range.

### TG analysis

Thermogravimetric (TG) analyses in argon (Ar 5.0) were run on the Netzsch TG 209 apparatus (range 298–973 K, Al_2_O_3_ crucible, empty crucible as a reference, a sample mass 8–10 mg, a heating rate 10 K min^−1^, a flow rate of the carrier gas 20 mL min^−1^).

### Potentiometric titrations

Potentiometric titrations were performed at 298.15 K by using Cerko Lab System microtitration unit fitted with 5‐mL Hamilton’s syringe and pH‐combined electrode (Schott‐BlueLine 16 pH type) All details for the measuring devices and the experimental setup were described in (Wyrzykowski et al. 2014). The ionic strength was maintained at 0.1 M using NaClO_4_. The 6 mM [bpyH][VO(nta)(H_2_O)]H_2_O and [phenH][VO(nta)(H_2_O)](H_2_O)_0.5_ solutions (V_o_ = 5.0 mL) were potentiometrically titrated with a standardized NaOH solution (0.098 M). The concentration distribution of various complex species existing in the solution as a function of pH was obtained using the HySS program (Alderighi et al. 1999).

### Cell culturing (hFOB, MG-63, HOS)

The cell lines: two human osteosarcoma cell lines (MG-63 and HOS) and untransformed hFOB were used to assess an anti-proliferative and cytotoxic effect, respectively. hFOB cells were grown in a mixture of Dulbecco’s Modified Eagle’s Medium and Ham F12 medium (1:1 ratio), MG-63 and HOS in Eagle’s Minimum Essential Medium also containing sodium pyruvate 110 mg/L and supplemented with 10% fetal bovine serum, 6 μg/mL penicillin-G, and 10 μg/mL streptomycin.

### Cell treatment (hFOB, MG-63, HOS)

Cultured cancer cells with 80–90% confluence were used for plating. The adherent cells were trypsinized to detach cells. 100 µL of cells were seeded into each well of the 96-wells plates (1–5 × 10^4^ cells per well). The plate was maintained at 37 °C in a incubator for 48 h until 80–90% confluence. Then, old media were discarded and the cells were treated with tested compounds and cisplatin as positive control. The concentrations of investigated compounds used in experiments were carefully selected according to the results obtained from a preliminary concentration–response study (data not shown). A stock solution of cisplatin was prepared by dissolving solid cisplatin in phosphate buffered saline (PBS; water solubility: 0.253 g/100 mL at 25 °C). Fresh solutions of cisplatin were made up for each experiment owing to its instability in water. The concentrations of cisplatin were selected based on published data found in the literature (Baharuddin et al. 2016; Křikavová et al. 2014). Both tested compounds and cisplatin were suspended in the SF cell culture and diluted to appropriate concentrations *ex tempore* every time before adding the cells. The dilutions of investigated compounds and cisplatin were filtered through a 0.22 µm membrane filter. Controls (negative) were treated with the serum free (SF) cell-culture medium.

## Results and discussion

### Chemical studies

#### The crystal structure description

**Fig. 4:**
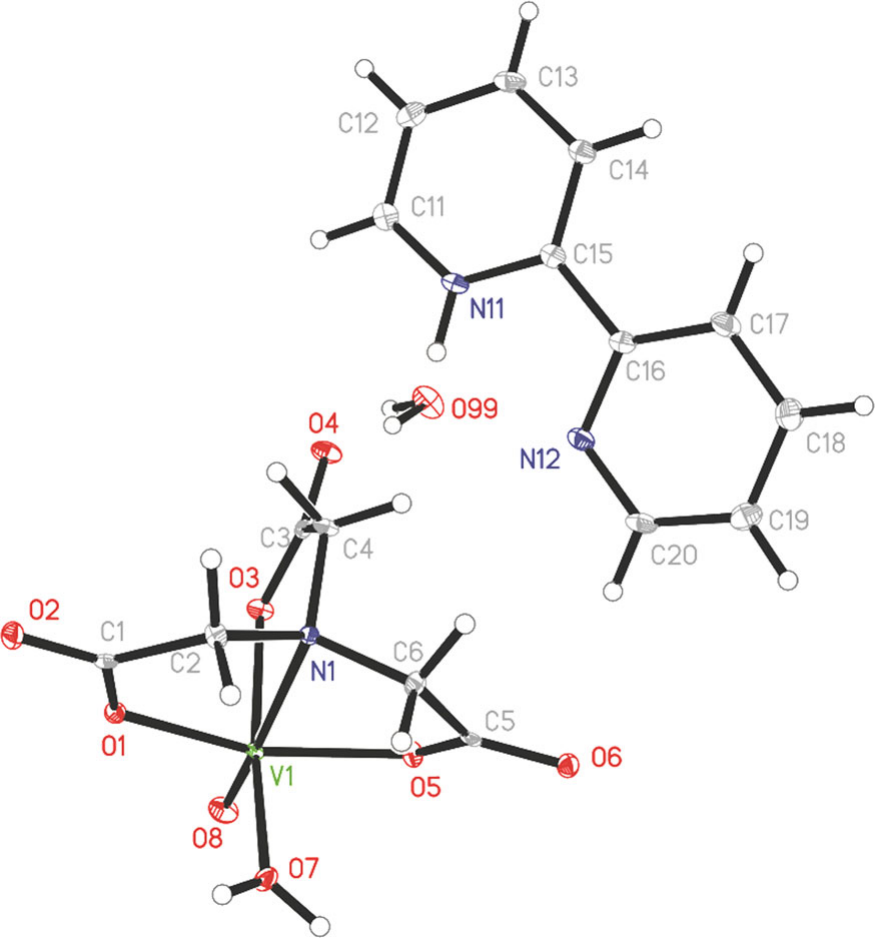
The molecular structure of [bpyH][VO(nta)(H_2_O)]H_2_O. Displacement ellipsoids are drawn at the 50% probability level, hydrogen atoms are drawn as spheres of arbitrary radii

A perspective view of the [bpyH][VO(nta)(H_2_O)]H_2_O structure together with the atom numbering scheme is shown in Fig. 4. All atoms of the compound lie in general positions and the asymmetric unit contains one 2,2′-bipyridinium cation, [bpyH]^+^, one [VO(nta)(H_2_O)]^−^ anion and one water molecule. The vanadium(IV) cation is six coordinated by three oxygen atoms and one nitrogen atom of the nta ligand, one oxygen atom of water molecule and one oxide ion. The coordination sphere of V(IV) adopts the geometry of distorted tetragonal bipyramid with the oxide and water oxygen atoms arranged in the cis geometry. The oxo ligand is located in the *trans* position to the nta nitrogen atom. The [V=O]^2+^ bond length (Table 2) agrees well with the average value of 1.600(1) Å resulting from the over 1000 corresponding structures deposited with the CCDC (Del Rio et al. 2003). The C–O bond lengths of nta (Table 2) confirm a one-and-a-half character of the bonds, caused by the delocalization of the π electrons of the carboxylate groups involved in the coordination of V(IV). The other vanadium–oxygen bond distances in the investigated compound are comparable with those found for its phenanthroline analogue, [phenH][VO(nta)(H_2_O)](H_2_O)_0.5_ (Tesmar et al. 2015).

**Table 2:** Selected structural data of [bpyH][VO(nta)(H2O)]H2O

Distance/angle	Å, °
V1—O8	1.5991 (10)
V1—O5	1.9927 (10)
V1—O1	1.9930 (10)
V1—O7	2.0181(10)
V1—O3	2.0250 (10)
V1—N1	2.3416 (11)
N11—C11	1.3415 (19)
N11—C15	1.3477 (18)
N12—C16	1.3463 (18)
N12—C20	1.3363 (19)
O1—C1	1.2876 (16)
O2—C1	1.2355 (17)
O3—C3	1.2804 (16)
O4—C3	1.2386 (17)
O5—C5	1.2792 (16)
O6—C5	1.2408 (17)
O8—V1—O5	104.02 (5)
O8—V1—O1	104.02 (5)
O5—V1—O1	150.45 (4)
O8—V1—O7	100.92 (5)
O5—V1—O7	85.47 (4)
O1—V1—O7	87.40 (4)
O8—V1—O3	94.47 (5)
O5—V1—O3	89.31 (4)
O1—V1—O3	90.03 (4)
O7—V1—O3	164.54 (4)
O8—V1—N1	171.60 (5)
O5—V1—N1	75.02 (4)
O1—V1—N1	76.04 (4)
O7—V1—N1	87.37 (4)
O3—V1—N1	77.22 (4)
C11—N11—C15	123.65 (12)
C20—N12—C16	117.27 (12)

The discrete mononuclear [VO(nta)(H_2_O)]^−^ coordination unit is unique among other known compounds containing nitrilotriacetate oxidovanadium(IV) moieties, namely tris(ammonium) μ-oxo-bis(nitrilotriacetato-oxo-vanadium) trihydrate (Nishizawa et al. 1979), catena-(bis(ammonium) bis(μ_2_-nitrilotriacetato)-(μ_2_-oxo)-tetra-aqua-dioxo-manganese-di-vanadium(IV) dihydrate), catena-(bis(ammonium) bis(μ_2_-nitrilotriacetato)-(μ_2_-oxo)-tetra-aqua-dioxo-di-vanadium(IV)-zinc dihydrate), nona-aqua-lanthanum ammonium (μ_2_-oxo)-bis(nitrilotriacetato)-dioxo-di-vanadium(IV), nona-aqua-neodymium ammonium (μ_2_-oxo)-bis(nitrilotriacetato)-dioxo-di-vanadium(IV) (Zhang et al. 2004), tripotassium (μ_2_-oxo)-bis((nitrilotriacetato)-oxo-vanadium(IV,V)) trihydrate (Shi et al. 2001) and ammonium non-aaqua-europium(III) (μ_2_-oxo)-bis(nitrilotriacetato)-dioxo-di-vanadium(IV) (Zhang et al. 2005), as typically these units are assembled to dimers via an oxide bridge. The dinuclear oxidovanadium(IV) coordination entities of the [(VO)_2_(μ_2_-O)(nta)_2_]^4−^ and [(VO)_2_(μ_2_-O)(nta)_2_M(H_2_O)_4_]^2−^ (M=Mn, Zn) types are formed with the inorganic cations (NH_4_^+^, La^3+^, Eu^3+^, Nd^3+^) acting as counter-ions. Recently, it has been proven in one specific case that the cation formed by a protonation of N-heterocyclic compound (i.e. phenH) is able to stabilize mononuclear [VO(nta)(H_2_O)]^−^ species (Tesmar et al. 2015). In this paper this finding is also confirmed for other protonated N-heterocyclic compound, i.e. 2,2′-bipyridinium cation, also as a counter-ion for the mononuclear oxidovanadium(IV) nitrilotriacetate anion. The vanadium–nitrogen bond distance in [bpyH][VO(nta)(H_2_O)]H_2_O (Table 2) is slightly longer than that found for the dinuclear [(VO)_2_(μ_2_-O)(nta)_2_]^4−^ coordination units (V–N 2.297(5) Å) (Zhang et al. 2004) as a result of presence of the water molecule in the inner coordination sphere of [VO(nta)(H_2_O)]^−^ instead of the bridging oxide ion.

All 2,2′-bipyridinium cation intramolecular distances and angles of the compound can be considered as normal for such cations. The elongation (and consequently weakening) of the C–N bonds (Table 2) formed by the protonated nitrogen atom (in comparison to non-protonated one) originates from transferring the electron density from the C-N bonds on the N–H bond. This phenomenon also affects the C–N–C angle, which is larger for the protonated nitrogen atom (Table 1). The pyridine rings of the [bpyH]^+^ cation are inclined at a dihedral angle of 9.37^o^. The V···V distance between neighbouring [VO(nta)(H_2_O)]^−^ (6.474 Å) is slightly shorter than that found for [phenH][VO(nta)(H_2_O)](H_2_O)_0.5_ (6.587 Å) as a result of the smaller volume of bpyH^+^ in comparison to phenH^+^. The [VO(nta)(H_2_O)]^−^ ions are linked through O–H···O hydrogen bonds (formed between the inner coordination sphere of water molecules and the oxygen atoms of the carboxylate groups, Table 3) to the folded ribbons extending along the crystallographic (010) axis and characterised by N_1_C(6)C(6)[N_2_R_2_^2^(12)] motifs of the graphs sets of a lowest degree.

**Table 3:** The hydrogen bonds geometry of [bpyH][VO(nta)(H_2_O)]H_2_O [Å, °]

D—H···A	d(D-H)	d(H···A)	d(D···A)	<(DHA)
O7—H7O···O2^i^	0.87	1.78	2.6492 (14)	172.2
O7—H7P···O6^vi^	0.84	1.75	2.5855 (14)	170.0
N11—H11 N···O4	0.92	2.00	2.7980 (15)	144.1
N11—H11 N···N12	0.92	2.23	2.6349 (17)	105.8
O99—H99O···O3^v^	0.91	2.02	2.9257 (14)	176.0
O99—H99P···O4	0.92	1.92	2.8253 (14)	167.9
C4—H4A···N12	0.99	2.67	3.5466 (18)	148.4
C6—H6A···O1^i^	0.99	2.66	3.3954 (16)	131.6
C6—H6B···O2^i^	0.99	2.61	3.3442 (16)	131.3
C14—H14···O99^iii^	0.95	2.60	3.5308 (19)	166.8
C17—H17···O99^iii^	0.95	2.57	3.4289 (18)	150.9
C18—H18···O8^vii^	0.95	2.33	3.1162 (18)	139.5
C19—H19···O1^iv^	0.95	2.57	3.4800 (17)	161.3
C20—H20···O6	0.95	2.41	3.2251 (17)	144.1

Symmetry transformations used to generate equivalent atoms: (i) −x + 1/2, y − 1/2, −z + 3/2; (ii) −x + 3/2, y − 1/2, −z + 3/2; (iii) −x + 2, −y + 1, −z + 1; (iv) x, y − 1, z; (v) − x + 1, − y + 2, −z + 1; (vi) −x + 1/2, y + 1/2, − z + 3/2; (vii) x + 1, y − 1, z

The outer coordination sphere of water molecules and [bpy(H)]^+^ cations is packed between the planes formed by parallel, above mentioned, folded ribbons (extending along crystallographic (010) plane). The O–H···O and N–H···O intermolecular hydrogen bonds link the outer coordination sphere species to the complex [VO(nta)(H_2_O)]^−^ anions (Table 3). All abovementioned interactions form the two-dimensional network extending along the crystallographic (101) plane. The neighbouring planes are expanded to the three-dimensional network via the weak C–H···O hydrogen bonds (Desiraju and Steiner 1999). Additionally the neighbouring bpyH^+^ cations are linked by π···π stacking interactions (Table 4) (Kruszyński and Sieranski 2016) to the dimers.

**Table 4:** Stacking interactions [Å, °]

R (I)···R (J)	Cg···Cg	α	β	d_p_
Cg (1)···Cg (1)^iii^	4.7663 (10)	0	45.05	−3.3672 (5)
Cg (1)···Cg (2)^iii^	3.7910 (9)	9.46 (7)	29.70	−3.5246 (5)
Cg (2)···Cg (1)^iii^	3.7909 (9)	9.46 (7)	21.61	−3.2928 (6)

Cg(1), Cg(2) indicates the centroids of six-membered aromatic rings (R) containing N11, N22 atoms respectively, α is a dihedral angle between planes I and J, β is an angle between Cg(I) and Cg(J) vector and normal to plane I and d_p_ is a perpendicular distance of Cg(I) on ring J plane

Symmetry transformations as in Table 3

### The IR spectroscopic characterization

The characteristic for the oxidovanadium(IV) compounds band at 981 cm^−1^ can be assigned to the V=O stretching mode (Pranczk et al. 2016; Banik et al. 2014). Two bands at 1586 and 1402 cm^−1^ correspond to the antisymmetric and symmetric vibrations of the ionized COO^−^ groups, respectively. This finding confirms the contribution of the carboxylate groups in the coordination of V(IV) in a monomeric [VO(nta)(H_2_O)]^−^ coordination entity. The difference, Δν, between the frequencies of asymmetrical [ν_as_(OCO^−^)] and symmetrical [ν_s_(OCO^−^)] vibrations for carboxylate group in the compound (Δν = 1586–1402 = 184 cm^−1^) and in the nitrilotriacetate sodium salt, Na_3_nta, Δν = 1598–1406 = 192 cm^−1^) suggests the ionic character of the VO-nta interactions (Nakamoto 2009). The band at 488 cm^−1^ corresponds to the stretching vibration *ν*(V–N) and agrees with the X-ray results showing that nta acts as a tetradentate ligand. The band at 1095 cm^−1^ that can be assigned to the stretching vibration *ν*(C–N) of the nta ligand (Tomita and Ueno 1963) is shifted ca. 100 cm^−1^ in relation to *ν*(C–N) in the free H_3_nta (1200 cm^−1^). It indicates that the N atom of the nta ligand coordinates to V atom. Most relevant infrared bands of bpyH^+^ are: 1474 cm^−1^ bpy − ν_ring_, 1456 cm^−1^ bpy − ν_ring_ + δ_ring-H_, 1274 cm^−1^, 1224 cm^−1^ and 1040 cm^−1^ bpy − δ(CH)_in plane_. The presence of the stretching vibration band at 3464 cm^−1^ indicates the attachment of a proton to the nitrogen atom of bpy. This is in line with the results obtained from the X-ray measurements. Moreover, the IR spectrum of the compound shows bands at 3300–3100 and 1660–1610 cm^−1^ that can be assigned to antisymmetric and symmetric OH stretching and HOH bending bands of the lattice and coordination water, respectively.

### The thermal analysis

The thermal decomposition of [bpyH][VO(nta)(H_2_O)]H_2_O proceeds in five main steps. The first two steps (115–160 and 160–185 °C, respectively) correspond to the loss of the lattice water (mass loss: found 3.9%, calcd. for H_2_O 4%) and one molecule of the coordination water (mass loss: found 4.3%, calcd. for H_2_O 4%). On further heating (above 185 °C) the compound undergoes a pyrolysis, which leads to the decomposition of the nta ligand in two overlapping steps. The last step (400–600 °C) is due to the loss of the remaining organic fragments (mainly bpy). In view of the overlapping processes which occur during the thermal decomposition of [bpyH][VO(nta)(H_2_O)]H_2_O it is difficult to suggest definite equations describing the process. The residual mass at 650 °C (ca 19%) can be assigned to the reduced, non-stoichiometric vanadium–oxygen phases. Under experimental conditions (the inert atmosphere, Ar) V(IV) can be reduced to V(III) and/or V(II) by an elemental carbon resulting as the product of the pyrolysis of the compound. The nitrogen atom of nta or bpy constitutes another factor that can participate in the inter- or/and intramolecular redox processes. Reducing properties of a nitrogen-containing ligands were also observed during thermal transformations of other coordination compounds (Ingier-Stocka and Bogacz 1989; Jacewicz et al. 2014).

### Solution studies

**Fig. 5:**
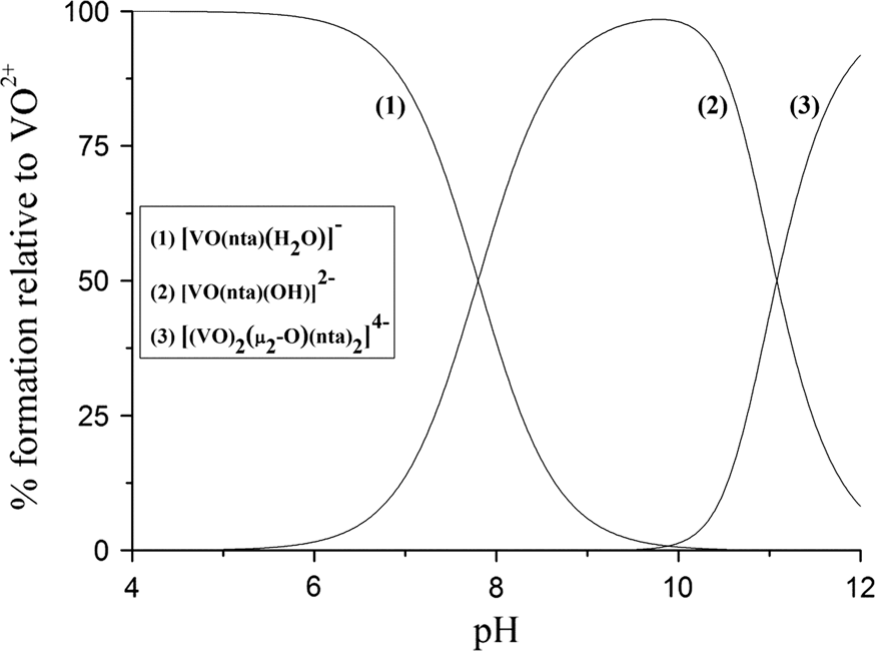
Species distribution curves of the VO(IV) species as a function of pH calculated based on the equilibrium constants for [bpyH][VO(nta)(H_2_O)] listed in Table 5

The potentiometric titration method has been applied for studying the stability of [bpyH][VO(nta)(H_2_O)]H_2_O and [phenH][VO(nta)(H_2_O)](H_2_O)_0.5_ in aqueous solutions. The equilibrium model that has given the best fit of the calculated data to the experimental ones is presented in Table 5. The logarithm of the overall equilibrium constants of the complex species (Table 5) were refined by least-squares calculations using the Hyperquad2008 (ver. 5.2.19) computer program (Gans et al. 1996). The representative species distribution diagram for [bpyH][VO(nta)(H_2_O)]H_2_O is displayed in Fig. 5.

**Table 5:** Logarithms of equilibrium constants of complex species at 298.15 K (standard deviation values in parentheses)

No.	Reaction	(bpyH) [VO(nta)(H_2_O)]	(phenH) [VO(nta)(H_2_O)]
1	A + H_3_O^+^ ${ \leftrightharpoons }$ AH^+^ + H_2_O AH^+^ denotes the 2,2′-bipyridinium (bpyH^+^) or 1,10–phenanthrolinium (phenH^+^) cation	4.47 (0.03)^a^	5.00 (0.04)^a^
2		7.80 (0.06)	7.93 (0.07)
3		−19.94 (0.06)	−19.91 (0.08)

^a^Literature data: the values of p*K*_a_ of bpyH^+^ and phenH^+^ are 4.52 (Jakusch et al. 2002) and 4.93 (Duma and Hancock 1994), respectively

Due to the presence of an aqua ligand in the coordination sphere of VO^2+^ the competition of the [VO(nta)(H_2_O)]^−^ ion with the organic cation (bpyH^+^ or phenH^+^) for hydroxide takes place. The aqua complex is stable to the pH of 5. At a higher pH range it undergoes a hydrolysis and the resulting hydroxo complex species ([VO(nta)(OH)]^2−^) reach the highest concentration at around pH 9.8. The ability of [VO(nta)(H_2_O)]^−^ to hydrolysis is a very important feature that has an impact on the susceptibility and a rate of the oxidation of (IV) to V(V) (Nishizawa et al. 1985). At the high concentration of the [VO(nta)(OH)]^2−^ ions the dinuclear species of the [(VO)_2_(μ_2_-O)(nta)_2_]^4−^ type are formed (Fig. 5). These type of oxidovanadium(IV) coordination entities have previously been reported in solid (Zhang et al. 2005). Thus, the similar coordination mode of the VO^2+^ cations can be expected in solutions. The oxo-bridged dioxidovanadium(IV) complexes ([(VO)_2_(μ_2_-O)(nta)_2_]^4−^) exist at equilibrium with the mononuclear [VO(nta)(OH)]^2−^ ions in aqueous solutions at pH above 10. Thus, physicochemical and biological properties of the nitrilotriacetate oxidovanadium(IV) ions are affected by the pH of the system under study.

### The cytotoxicity of oxidovanadium(IV) compounds in human osteoblast and osteosarcoma cell lines

#### The cytotoxicity of the compounds

**Fig. 6:**
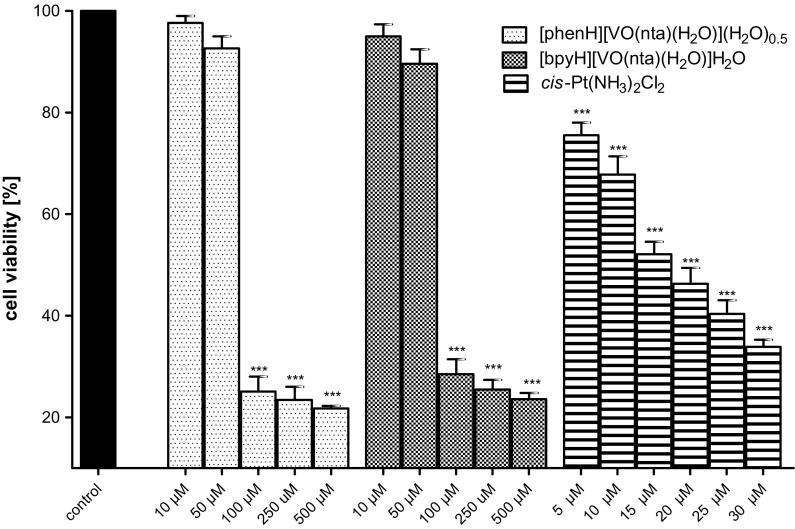
The viability of the human osteoblast cell line (hFOB 1.10) detected by the LDH test after 48 h of an exposure to investigated compounds and cisplatin (as a positive control). Data are expressed as mean values ± SD from three experiments. ***p < 0.001 versus control

**Fig. 7:**
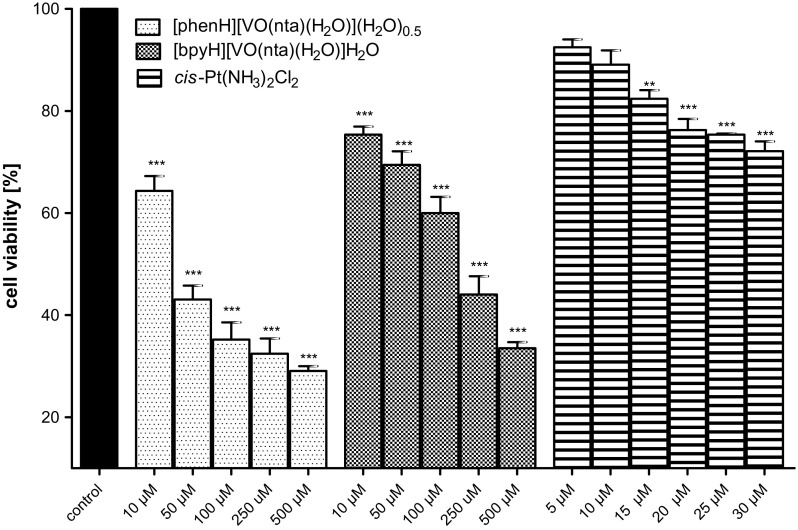
The viability of the human osteosarcoma cell line (MG-63) assessed by the LDH test after 48 h of an exposure to investigated compounds and cisplatin (as a positive control). Data are expressed as mean values ± SD from three experiments. **p < 0.01; ***p < 0.001 versus control

The concentration-dependent effects of investigated compounds on the normal, hFOB (hFOB 1.10) and human osteosarcoma cell line (MG-63) were tested at the plasma membrane level (the LDH leakage) after 48 h of an incubation (Figs. 6, 7). The results were referred to the aqueous soluble inorganic derivative of bi-valent platinum, i.e. cisplatin (*cis*-Pt(NH_3_)_2_Cl_2_) (Florea and Busselberg 2011; Prylutskyy et al. 2015). Cisplatin is currently one of the most extensively used chemotherapeutic drugs for the cancer treatment (Leon et al. 2014a, b). In osteosarcoma cells *cis*-Pt(NH_3_)_2_Cl_2_ induces a selective inhibition of DNA synthesis and, as a consequence, a cell proliferation and reproduction.

In the concentration range of 10–50 μM no significant cytotoxic effects of the compounds on the untransformed hFOB (hFOB 1.19) were observed. The highest concentration of the compounds (100–500 μM) triggers a decrease in the viability of the hFOB 1.19 cells to about 20%. As far as the human osteosarcoma cell line (MG-63) is concerned, the concentration-dependent cytotoxic effect of the compounds was observed. It is interesting to note that both compounds exhibited a stronger cytotoxicity than cisplatin used as a positive control. The study has revealed that the compounds, in the low concentration range (10–50 μM), have a significant selectivity for malignant cells. These results point to the fact that the investigated compounds show promising properties to be further investigated as possible antitumor agents in this model of bone-related cells.

### The antiproliferative activity of the compounds

**Fig. 8:**
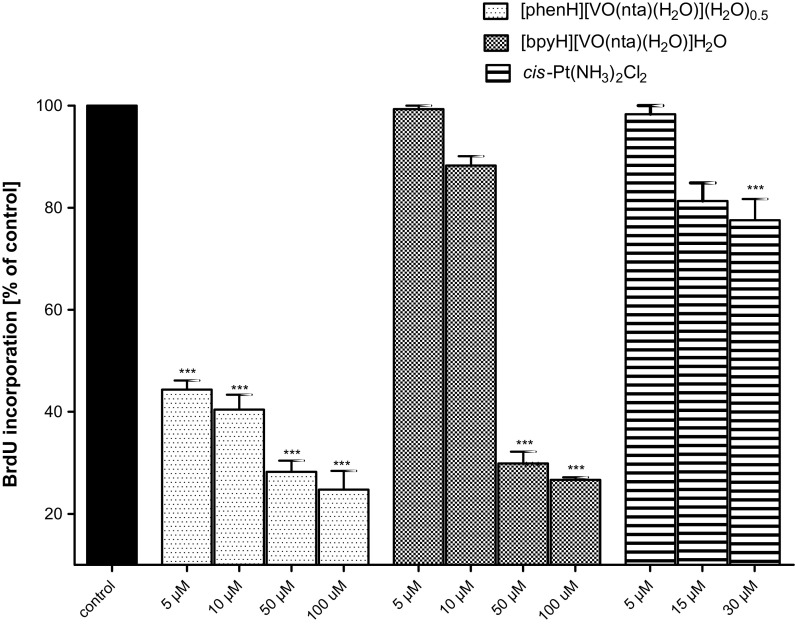
The inhibition of the human osteosarcoma cell (MG-63) proliferation after an incubation with investigated compounds assessed with the BrdU-test. Cells were incubated with increasing concentrations of the tested compounds and cisplatin (as a positive control) for 48 h. Data are expressed as mean values ± SD from three experiments. ***p < 0.001 versus control

**Fig. 9:**
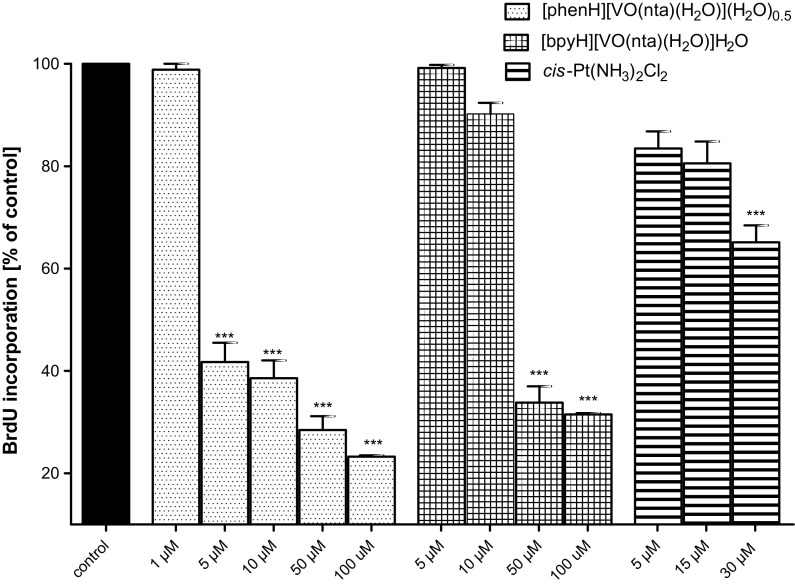
The inhibition of the human osteosarcoma cell (HOS) proliferation after an incubation with investigated compounds assessed with the BrdU-test. Cells were incubated with increasing concentrations of the tested compounds and cisplatin (as a positive control) for 48 h. Data are expressed as mean values ± SD from three experiments. ***p < 0.001 versus control

Many attempts have been taken to determine the putative mechanisms of an action involved in the antitumoral effects of the oxidovanadium(IV) compounds (Rivadeneira et al. 2010; Leon et al. 2013, 2014a, 2015, 2016a, b; Ferrer et al. 2006). It has been reported that these compounds, depending on the cellular line, may promote the generation of the reactive oxygen species (ROS) mainly in the mitochondria (Leon et al. 2012a, 2014b; Rivadeneira et al. 2009; Di Virgilio et al. 2011) leading to a decrease of glutathione (GSH) concentration. GSH is one of the mayor reducing agents responsible for maintaining the cellular redox status through the balance of the couple glutathione/glutathione disulphide (GSH/GSSG). The depletion of GSH concentration alters the intracellular redox balance (GSH/GSSG) on an account of the accumulation of GSSG inside the cells. Furthermore, the oxidative stress causes a dissipation of the mitochondria membrane potential (MMP) that can lead the cells into apoptosis and necrosis (Mayer and Oberbauer 2003). The concentration-dependent (in the concentration range of 1–100 μM) effect of the compounds on the two human MG-63 and HOS osteosarcoma cell lines was investigated by the measurement of the BrdU (5-bromo-2′-deoxyuridine) incorporation by actively dividing cells after 48 h of culture in the presence of different concentrations of compounds (Figs. 8, 9). It has been found that all investigated compounds induce a significant reduction of the BrdU incorporation into cellular DNA indicating a concentration-dependent anti-proliferative effect. Importantly, the cytotoxic effect of the compounds against cancer cell line was already found at the concentrations which are non-toxic for untransformed hFOB. Based on the obtained results it has been found that [phenH][VO(nta)(H_2_O)](H_2_O)_0.5_ exhibit the most effective anti-proliferative activity towards MG-63 and HOS cell lines. It is interesting to note that its anti-proliferative activity is significantly higher than that found for *cis*-Pt(NH_3_)_2_Cl_2_. The high anti-proliferative activity of [phenH][VO(nta)(H_2_O)](H_2_O)_0.5_ can be assigned to the intercalating properties of the phen derivative that interacts more strongly with DNA than the bpy derivative. A similar relationship in the reactivity towards DNA has also been found for other phen and bpy metal coordination compounds (Yodoshi et al. 2007; Chakravarty 2006).

## Conclusions

When the inorganic cations (NH_4_^+^, La^3+^, Eu^3+^, Nd^3+^) are present as the counter-ions, mononuclear [VO(nta)(H_2_O)]^−^ coordination units easily undergo a dimerization via an oxide bridge. We have previously reported that the use of the cation formed by protonation of N-heterocyclic compound (i.e. 1,10–phenanthrolinium cation, phenH^+^) enabled to obtain discrete mononuclear [VO(nta)(H_2_O)]^−^ coordination ions. This finding has been confirmed in the present study for other protonated N-heterocyclic compound, i.e. 2,2′-bipyridinium cation, [bpyH^+^]. The X-ray measurements have revealed that [bpyH][VO(nta)(H_2_O)]H_2_O comprises the discrete mononuclear [VO(nta)(H_2_O)]^−^ coordination ions linked through O–H···O hydrogen bonds formed between the inner coordination sphere of water molecules and the oxygen atoms of the carboxylate groups. IR spectra as well as thermal analysis have confirmed the results obtained from the X-ray measurements.

The potentiometric titration method has successfully been applied to assess the stability of the complexes in aqueous solutions. The [VO(nta)(H_2_O)]^−^ ion undergoes a hydrolysis. At a higher pH range (above pH 10) the resulting hydroxo complexes form the oxo-bridged dioxidovanadium(IV) species of the [(VO)_2_(μ_2_-O)(nta)_2_]^4−^ type. Thus, the physicochemical properties and biological activities of the complexes depend on the pH of the investigated system.

Biological studies (the MTT and LDH tests) have proven that [bpyH][VO(nta)(H_2_O)]H_2_O and [phenH][VO(nta)(H_2_O)](H_2_O)_0.5_ show promising antitumor activity towards human osteosarcoma cell lines (MG-63 and HOS). In the low concentration range they exert a stronger cytotoxic effect on osteosarcoma cells than in the untransformed human osteoblast cells. [phenH][VO(nta)(H_2_O)](H_2_O)_0.5_ exhibit a higher anti-proliferative activity towards MG-63 and HOS than [bpyH][VO(nta)(H_2_O)]H_2_O and *cis*-Pt(NH_3_)_2_Cl_2_ (as a positive control). A significant selectivity of the compounds under study for malignant cells suggest that the [VO(nta)(H_2_O)]^−^ containing compounds constitute an important group of compounds which are worth to consider as possible antitumor agents in the osteosarcoma model of bone-related cells in culture.

## Electronic supplementary material

Below is the link to the electronic supplementary material.

Supplementary material 1 (PDF 120 kb)

## References

[CR1] Alderighi L, Gans P, Ienco A, Peters D, Sabatini A, Vacca A (1999). Hyperquad simulation and speciation (HySS): a utility program for the investigation of equilibria involving soluble and partially soluble species. Coord Chem Rev.

[CR2] Baharuddin P, Satar N, Fakiruddin KS, Zakaria N, Lim MN, Yusoff NM, Zakaria Z, Yahaya BH (2016). Curcumin improves the efficacy of cisplatin by targeting cancer stem-like cells through p21 and cyclin D1-mediated tumour cell inhibition in non-small cell lung cancer cell lines. Oncol Rep.

[CR3] Banik B, Somyajit K, Nagaraju G, Chakravarty AR (2014). Oxovanadium(IV) complexes of curcumin for cellular imaging and mitochondria targeted photocytotoxicity. Dalton Trans.

[CR4] Chakravarty AR (2006). Photocleavage of DNA by copper(II) complexes. J Chem Sci.

[CR5] D’Cruz OJ, Uckun FM (2002). Metvan: a novel oxovanadium(IV) complex with broad spectrum anticancer activity. Expert Opin Investig Drugs.

[CR6] Del Rio D, Galindo A, Vicente R, Mealli C, Ienco A, Masi D (2003). Synthesis, molecular structure and properties of oxo-vanadium(IV) complexes containing the oxydiacetate ligand. Dalton Trans.

[CR7] Desiraju GR, Steiner T (1999). The weak hydrogen bond in structural chemistry and biology.

[CR8] Di Virgilio AL, Rivadeneira J, Muglia CI, Reigosa MA, Butenko N, Cavaco I, Etcheverry SB (2011). Cyto-and genotoxicity of a vanadyl(IV) complex with oxodiacetate in human colon adenocarcinoma (Caco-2) cells: potential use in cancer therapy. Biometals.

[CR9] Dong Y, Narla RK, Sudbeck E, Uckun FM (2000). Synthesis, X-ray structure, and anti-leukemic activity of oxovanadium(IV) complexes. J Inorg Biochem.

[CR10] Duma TW, Hancock RD (1994). The affinity of the vanadyl(IV) ion for nitrogen donor ligands. J Coord Chem.

[CR11] Evangelou AM (2002). Vanadium in cancer treatment. Crit Rev Oncol Hematol.

[CR12] Felcman J, Fraústo da Silva JJR (1983). Complexes of oxovanadium(IV) with polyaminocarboxylic acids. Talanta.

[CR13] Ferrer EG, Salinas MV, Correa MJ, Naso L, Barrio DA, Etcheverry SB, Lezama L, Rojo T, Williams PAM (2006). Synthesis, characterization, antitumoral and osteogenic activities of quercetin vanadyl(IV) complexes. J Biol Inorg Chem.

[CR14] Florea AM, Busselberg D (2011). Cisplatin as an anti-tumor drug: cellular mechanisms of activity, drug resistance and induced side effects. Cancer.

[CR15] Gambino D (2011). Potentiality of vanadium compounds as anti-parasitic agents. Coord Chem Rev.

[CR16] Gans P, Sabatini A, Vacca A (1996). Investigation of equilibria in solution. Determination of equilibrium constants with the HYPERQUAD suite of programs. Talanta.

[CR17] Gleeson B, Claffey J, Deally A, Hogan M, Méndez LMM, Müller-Bunz H, Patil S, Wallis D, Tacke M (2009). Synthesis and cytotoxicity studies of fluorinated derivatives of vanadocene Y. Eur J Inorg Chem.

[CR18] Harding CJ, Henderson RK, Powell AK (1993). A new type of hexanuclear iron(III) hydroxo(oxo) cluster. Angew Chem Int Ed Engl.

[CR19] Havelek R, Siman P, Cmielova J, Stoklasova A, Vavrova J, Vinklarek J, Knizek J, Rezacova M (2012). Differences in vanadocene dichloride and cisplatin effect on MOLT-4 leukemia and human peripheral blood mononuclear cells. Med Chem.

[CR20] Ingier-Stocka E, Bogacz A (1989). Thermal decomposition of [Co (NH3) 6] Cl3: part I. Non-isothermal, quasi-isothermal and scanning electron microscopy studies. J Therm Anal Calorim.

[CR21] Jacewicz D, Pranczk J, Wyrzykowski D, Żamojć K, Chmurzyński L (2014). Thermal properties of [Co(en)_2_Cl_2_]Cl in solid state. Cis–trans isomerization of the [Co(en)_2_Cl_2_]^+^ complex ion in methanol. React Kinet Mech Catal.

[CR22] Jakusch T, Buglyó P, Tomaz AI, Pessoa JC, Kiss T (2002). Thiolate-S as anchoring donor in the binary and ternary VO(IV) complexes of mercaptopropionylglycine. Inorg Chim Acta.

[CR23] Jakusch T, Pessoa JC, Kiss T (2011). The speciation of vanadium in human serum. Coord Chem Rev.

[CR24] Kioseoglou E, Petanidis S, Gabriel C, Salifoglou A (2015). The chemistry and biology of vanadium compounds in cancer therapeutics. Coord Chem Rev.

[CR25] Křikavová R, Hošek J, Vančo J, Hutyra J, Dvořák Z, Trávníček Z (2014). Gold(I)–triphenylphosphine complexes with hypoxanthine-derived ligands. In vitro evaluations of anticancer and anti-inflammatory activities. PLoS ONE.

[CR26] Kruszyński R, Sieranski T (2016). Can stacking interactions exist beyond the commonly accepted limits?. Cryst Growth Des.

[CR27] Leon IE, Di Virgilio AL, Barrio DA, Arrambide G, Gambino D, Etcheverry SB (2012). Hydroxylamido–amino acid complexes of oxovanadium(V). Toxicological study in cell culture and in a zebrafish model. Metallomics.

[CR28] Leon IE, Etcheverry SB, Parajón-Costa BS, Baran EJ (2012). Spectroscopic characterization of an oxovanadium(IV) complex of oxodiacetic acid and o-phenanthroline. Bioactivity on osteoblast-like cells in culture. Biol Trace Elem Res.

[CR29] Leon IE, Di Virgilio AL, Porro V, Muglia CI, Naso LG, Williams PAM, Bollati-Fogolin M, Etcheverry SB (2013). Antitumor properties of a vanadyl(IV) complex with the flavonoid chrysin [VO(chrysin)_2_EtOH]_2_ in a human osteosarcoma model: the role of oxidative stress and apoptosis. Dalton Trans.

[CR30] Leon IE, Porro V, Di Virgilio AL, Naso LG, Williams PAM, Bollati-Fogolín M, Etcheverry SB (2014). Antiproliferative and apoptosis-inducing activity of an oxidovanadium(IV) complex with the flavonoid silibinin against osteosarcoma cells. J Biol Inorg Chem.

[CR31] Leon IE, Butenko N, Di Virgilio AL, Muglia CI, Baran EJ, Cavaco I, Etcheverry SB (2014). Vanadium and cancer treatment: antitumoral mechanisms of three oxidovanadium(IV) complexes on a human osteosarcoma cell line. J Inorg Biochem.

[CR32] Leon IE, Cadavid-Vargas JF, Tiscornia I, Porro V, Castelli S, Katkar P, Desideri A, Bollati-Fogolin M, Etcheverry SB (2015). Oxidovanadium(IV) complexes with chrysin and silibinin: anticancer activity and mechanisms of action in a human colon adenocarcinoma model. J Biol Inorg Chem.

[CR33] Leon IE, Cadavid-Vargas JF, Resasco A, Maschi F, Ayala MA, Carbone C, Etcheverry SB (2016). In vitro and in vivo antitumor effects of the VO–chrysin complex on a new three-dimensional osteosarcoma spheroids model and a xenograft tumor in mice. J Biol Inorg Chem.

[CR34] Leon IE, Cadavid-Vargas JF, Di Virgilio AL, Etcheverry SB (2016). Vanadium, ruthenium and copper compounds: a new class of non-platinum metallodrugs with anticancer activity. Curr Med Chem.

[CR35] Levina A, Lay PA (2011). Metal-based anti-diabetic drugs: advances and challenges. Dalton Trans.

[CR36] Marzban L, McNeill JH (2003). Insulin-like actions of vanadium: potential as a therapeutic agent. J Trace Elem Exp Med.

[CR37] Marzo T, Bartoli G, Gabbiani C, Pescitelli G, Severi M, Pillozzi S, Michelucci E, Fiorini B, Arcangeli A, Quiroga AG, Messori L (2016). Cisplatin and its dibromido analogue: a comparison of chemical and biological profiles. Biometals.

[CR38] Mayer B, Oberbauer R (2003). Mitochondrial regulation of apoptosis. News Physiol Sci.

[CR39] McNeill JH, Yuen VG, Hoveyda HR, Orvig C (1992). Bis(maltolato)oxovanadium(IV) is a potent insulin mimic. J Med Chem.

[CR40] Nakamoto K (2009). Infrared and Raman spectra of inorganic and coordination compounds part B: applications in coordination, organometallic, and bioinorganic chemistry.

[CR41] Nishizawa M, Hirotsu K, Ooi S, Saito K (1979). A mixed valence binuclear complex of vanadium(IV) and vanadium(V). X-Ray crystal structure of (NH_4_)_3_[V_2_O_3_(nitrotriacetate)_2_]·3H_2_O. J Chem Soc Chem Commun.

[CR42] Nishizawa M, Sasaki Y, Saito K (1985). Kinetics and mechanisms of the outer-sphere oxidation of cis-aquaoxovanadium(IV) complexes containing quadrindentate amino polycarboxylates. Interpretation of the difference in activation parameters with the charge type of reactants. Inorg Chem.

[CR43] Palackova H, Vinklarek J, Holubova J, Cisarova I, Erben M (2007). The interaction of antitumor active vanadocene dichloride with sulfur-containing amino acids. J Organomet Chem.

[CR44] Pessoa JC, Tomaz I (2010). Transport of therapeutic vanadium and ruthenium complexes by blood plasma components. Curr Med Chem.

[CR45] Pessoa JC, Garribba E, Santos MF, Santos-Silva T (2015). Vanadium and proteins: uptake, transport, structure, activity and function. Coord Chem Rev.

[CR46] Pessoa JC, Etcheverry S, Gambino D (2015). Vanadium compounds in medicine. Coord Chem Rev.

[CR47] Pranczk J, Tesmar A, Wyrzykowski D, Inkielewicz-Stępniak I, Jacewicz D, Chmurzyński L (2016). Influence of primary ligands (ODA, TDA) on physicochemical and biological properties of oxidovanadium(IV) complexes with bipy and phen as auxiliary ligands. Biol Trace Elem Res.

[CR48] Prylutskyy YI, Cherepanov VV, Evstigneev MP, Kyzyma OA, Petrenko VI, Styopkin VI, Bulavin LA, Davidenko NA, Wyrzykowski D, Woziwodzka A, Piosik J, Kaźmierkiewicz R, Ritter U (2015). Structural self-organization of C_60_ and cisplatin in physiological solution. Phys Chem Chem Phys.

[CR49] Rehder D (2013). The future of/for vanadium. Dalton Trans.

[CR50] Rehder D (2017). Implications of vanadium in technical applications and pharmaceutical issues. Inorg Chim Acta.

[CR51] Rivadeneira J, Barrio DA, Arrambide G, Gambino D, Bruzzone L, Etcheverry SB (2009). Biological effects of a complex of vanadium(V) with salicylaldehyde semicarbazone in osteoblasts in culture: mechanism of action. J Inorg Biochem.

[CR52] Rivadeneira J, Di Virgilio AL, Barrio DA, Muglia CI, Bruzzone L, Etcheverry SB (2010). Cytotoxicity of a vanadyl(IV) complex with a multidentate oxygen donor in osteoblast cell lines in culture. Med Chem.

[CR53] Sheldrick GM (2003) SADABS. University of Gottingen, Germany

[CR54] Sheldrick GM (2015). SHELXT—integrated space-group and crystal-structure determination. Acta Crystallogr A.

[CR55] Sheldrick GM (2015). Crystal structure refinement with SHELXL. Acta Crystallogr C.

[CR56] Shi JM, Xu JQ, Yu WT, Liu LD, Wu CJ (2001). Synthesis, crystal structure and magnetism of vanadium(IV/V) complex: K_3_[V_2_O_3_(nta)_2_] × 3H_2_O. Pol J Chem.

[CR57] Shukla R, Barve V, Padhye S, Bhonde R (2006). Reduction of oxidative stress induced vanadium toxicity by complexing with a flavonoid, quercetin: a pragmatic therapeutic approach for diabetes. Biometals.

[CR58] Srivastava AK, Mehdi MZ (2005). Insulino-mimetic and anti-diabetic effects of vanadium compounds. Diabet Med.

[CR59] Tesmar A, Inkielewicz-Stępniak I, Sikorski A, Wyrzykowski D, Jacewicz D, Zięba P, Pranczk J, Ossowski T, Chmurzyński L (2015). Structure, physicochemical and biological properties of new complex salt of aqua-(nitrilotriacetato-N, O, O′, O″)-oxidovanadium(IV) anion with 1,10-phenanthrolinium cation. J Inorg Biochem.

[CR60] Thompson KH, Orvig C (2006). Vanadium in diabetes: 100 years from phase 0 to phase I. J Inorg Biochem.

[CR61] Thompson KH, Lichter J, LeBel C, Scaife MC, McNeill JH, Orvig C (2009). Vanadium treatment of type 2 diabetes: a view to the future. J Inorg Biochem.

[CR62] Tomita Y, Ueno K (1963). The properties and infrared absorption spectra of nitrilotriacetate chelates. Bull Chem Soc Jpn.

[CR63] Vinklarek J, Honzıcek J, Holubova J (2004). Interaction of the antitumor agent vanadocene dichloride with phosphate buffered saline. Inorg Chim Acta.

[CR64] Willsky GR, Chi LH, Godzala M, Kostyniak PJ, Smee JJ, Trujillo AM, Alfano JA, Ding WJ, Hu ZH, Crans DC (2011). Anti-diabetic effects of a series of vanadium dipicolinate complexes in rats with streptozotocin-induced diabetes. Coord Chem Rev.

[CR65] Wyrzykowski D, Inkielewicz-Stępniak I, Czupryniak J, Jacewicz D, Ossowski T, Woźniak M, Chmurzyński L (2013). Electrochemical and biological studies on reactivity of [VO(oda)(H_2_O)_2_],[Co(oda)(H_2_O)_2_]·H_2_O, and [Ni (oda)(H_2_O)_3_]·1.5 H_2_O towards superoxide free radicals. Z Anorg Allg Chem.

[CR66] Wyrzykowski D, Tesmar A, Jacewicz D, Pranczk J, Chmurzyński L (2014). Zinc(II) complexation by some biologically relevant pH buffers. J Mol Recognit.

[CR67] Wyrzykowski D, Inkielewicz-Stępniak I, Pranczk J, Żamojć K, Zięba P, Tesmar A, Jacewicz D, Ossowski T, Chmurzyński L (2015). Physicochemical properties of ternary oxovanadium(IV) complexes with oxydiacetate and 1,10-phenanthroline or 2,2′-bipyridine. Cytoprotective activity in hippocampal neuronal HT22 cells. Biometals.

[CR68] Wyrzykowski D, Kloska A, Pranczk J, Szczepańska A, Tesmar A, Jacewicz D, Pilarski B, Chmurzyński L (2015). Physicochemical and biological properties of oxovanadium(IV), cobalt(II) and nickel(II) complexes with oxydiacetate anions. Biol Trace Elem Res.

[CR69] Yodoshi M, Odoko M, Okabe N (2007). Structures and DNA-binding and cleavage properties of ternary copper(II) complexes of glycine with phenanthroline, bipyridine, and bipyridylamine. Chem Pharm Bull.

[CR70] Zhang Q, Lu C, Yang W, Chen S, Yu Y, He X, Yan Y, Liu J, Xu X, Xia C, Chen L, Wu X (2004). Synthesis and characterization of vanadium(IV)–M (M = Mn, Zn) and vanadium(IV)–Ln (Ln = La, Nd) complexes with nitrilotriacetate ligands: {(NH_4_)_2_[(V^IV^O)_2_(µ_2_-O)(nta)_2_M(H_2_O)_4_]·2H_2_O}_n_ and NH_4_[Ln(H_2_O)_9_][(V^IV^O)_2_ (µ_2_-O)(nta)_2_]. Polyhedron.

[CR71] Zhang QZ, Chen SM, Yu YQ, He X, Yan Y, Liu JH, Xu XJ, Xia CK, Chen LJ, Wu XY, Lu CZ (2005). Synthesis and crystal structure of a binuclear vanadium complex: NH_4_[(V^IV^O)_2_(μ_2_-O)(nta)_2_] [EuIII(H_2_O)_9_]. Chin J Inorg Chem.

