# Supplementary material for: Characterization and cytotoxic effect of aqua-(2,2′,2′′-nitrilotriacetato)-oxo-vanadium salts on human osteosarcoma cells
Source: Biometals. 2017 Feb 15;30(2):261–75. doi: 10.1007/s10534-017-0001-6 (PMC5352783; doi:10.1007/s10534-017-0001-6)

checkCIF/PLATON report

Structure factors have been supplied for datablock(s) gda115

THIS REPORT IS FOR GUIDANCE ONLY. IF USED AS PART OF A REVIEW PROCEDURE FOR PUBLICATION, IT SHOULD NOT REPLACE THE EXPERTISE OF AN EXPERIENCED CRYSTALLOGRAPHIC REFEREE.

No syntax errors found. CIF dictionary Interpreting this report

Datablock: gda115

Bond precision:	C-C = 0.0019 A	Wavelength=1.54178	
Cell:	a=7.3532(6)	b=9.6573(13)	c=25.403(3)
	alpha=90	beta=90.678(9)	gamma=90
Temperature:	100 K		

	Calculated	Reported
Volume	1803.8(4)	1803.8(4)
Space group	P 21/n	P 21/n
Hall group	-P 2yn	-P 2yn
Moiety formula	C6 H8 N O8 V, C10 H9 N2, H2 O	C6 H8 N O8 V 1-, C10 H9 N2 1+, H2 O
Sum formula	C16 H19 N3 O9 V	C16 H19 N3 O9 V
Mr	448.28	448.28
Dx,g cm-3	1.651	1.651
Z	4	4
Mu (mm-1)	5.146	5.146
F000	924.0	924.0
F000’	927.48	
h,k,lmax	9,11,31	9,11,31
Nref	3554	3547
Tmin,Tmax	0.566,0.571	0.571,0.598
Tmin’	0.514	

Correction method= # Reported T Limits: Tmin=0.571 Tmax=0.598
AbsCorr = MULTI-SCAN

Data completeness= 0.998 Theta(max)= 72.401

R(reflections)= 0.0255(3546) wR2(reflections)= 0.0674(3547)

S = 1.064 Npar= 263

```
The following ALERTS were generated. Each ALERT has the format
       test-name_ALERT_alert-type_alert-level.
Click on the hyperlinks for more details of the test.
```

Alert level C

```
PLAT911_ALERT_3_C Missing # FCF Refl Between THmin & STh/L=  0.600          3 Report
```

Alert level G

```
PLAT007_ALERT_5_G Number of Unrefined Donor-H Atoms .............           5 Report
PLAT232_ALERT_2_G Hirshfeld Test Diff (M-X)  V1     --  O8      ..        7.2 s.u.
PLAT232_ALERT_2_G Hirshfeld Test Diff (M-X)  V1     --  N1      ..        8.6 s.u.
PLAT912_ALERT_4_G Missing # of FCF Reflections Above STh/L=  0.600          5 Note
PLAT961_ALERT_5_G Dataset Contains no Negative Intensities .......     Please Check
PLAT978_ALERT_2_G Number C-C Bonds with Positive Residual Density          12 Note
```

```
   0 ALERT level A = Most likely a serious problem - resolve or explain
   0 ALERT level B = A potentially serious problem, consider carefully
   1 ALERT level C = Check. Ensure it is not caused by an omission or oversight
   6 ALERT level G = General information/check it is not something unexpected

   0 ALERT type 1 CIF construction/syntax error, inconsistent or missing data
   3 ALERT type 2 Indicator that the structure model may be wrong or deficient
   1 ALERT type 3 Indicator that the structure quality may be low
   1 ALERT type 4 Improvement, methodology, query or suggestion
   2 ALERT type 5 Informative message, check
```

It is advisable to attempt to resolve as many as possible of the alerts in all categories. Often the minor alerts point to easily fixed oversights, errors and omissions in your CIF or refinement strategy, so attention to these fine details can be worthwhile. In order to resolve some of the more serious problems it may be necessary to carry out additional measurements or structure refinements. However, the purpose of your study may justify the reported deviations and the more serious of these should normally be commented upon in the discussion or experimental section of a paper or in the "special_details" fields of the CIF. checkCIF was carefully designed to identify outliers and unusual parameters, but every test has its limitations and alerts that are not important in a particular case may appear. Conversely, the absence of alerts does not guarantee there are no aspects of the results needing attention. It is up to the individual to critically assess their own results and, if necessary, seek expert advice.

Publication of your CIF in IUCr journals

A basic structural check has been run on your CIF. These basic checks will be run on all CIFs submitted for publication in IUCr journals (*Acta Crystallographica*, *Journal of Applied Crystallography*, *Journal of Synchrotron Radiation*); however, if you intend to submit to *Acta Crystallographica Section C* or *E* or *IUCrData*, you should make sure that full publication checks are run on the final version of your CIF prior to submission.

Publication of your CIF in other journals

Please refer to the *Notes for Authors* of the relevant journal for any special instructions relating to CIF submission.

PLATON version of 06/05/2016; check.def file version of 05/05/2016

Datablock gda115 - ellipsoid plot

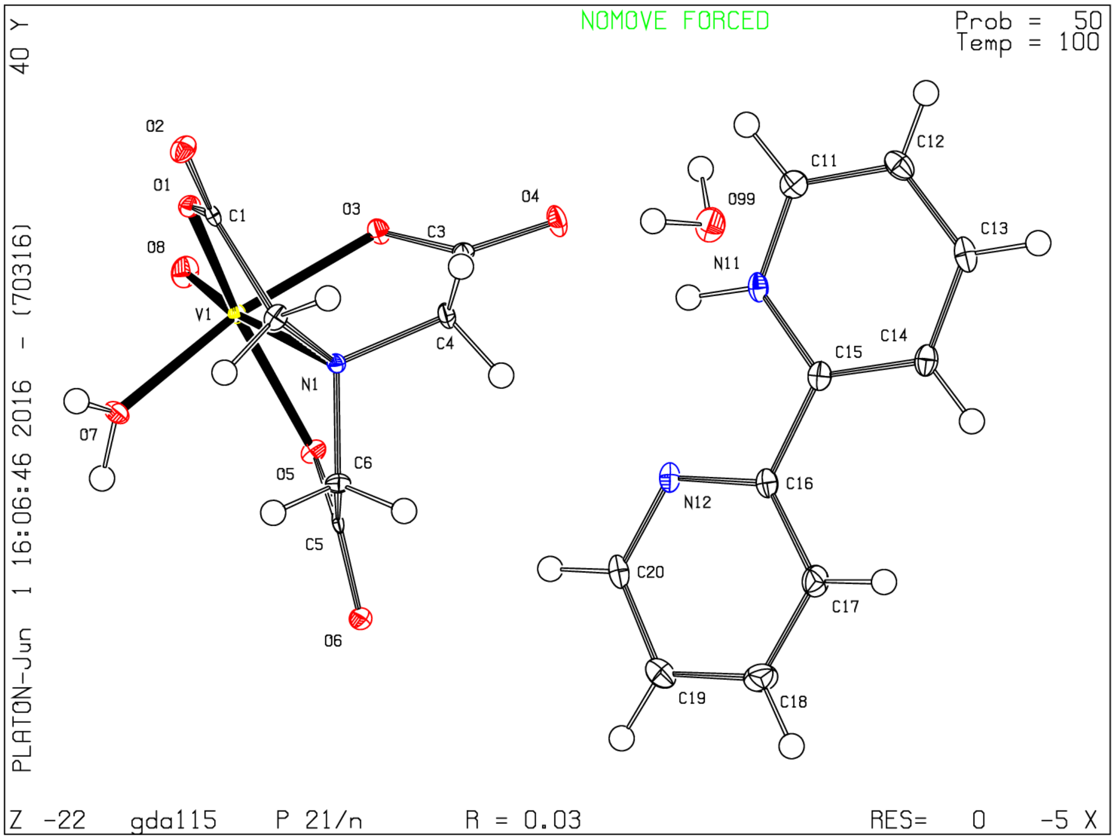

Supplement: Supplementary file 1 — Supplementary material 1 (PDF 120 kb) [file 10534_2017_1_MOESM1_ESM.pdf]
